# Wireless Cortical Brain-Machine Interface for Whole-Body Navigation in Primates

**DOI:** 10.1038/srep22170

**Published:** 2016-03-03

**Authors:** Sankaranarayani Rajangam, Po-He Tseng, Allen Yin, Gary Lehew, David Schwarz, Mikhail A. Lebedev, Miguel A. L. Nicolelis

**Affiliations:** 1 Department of Neurobiology, Duke University Medical Center, Durham, NC; 2 Duke Center for Neuroengineering, Duke University, Durham, NC; 3 Department of Biomedical Engineering, Duke University, Durham, NC; 4 Department of Psychology and Neuroscience, Duke University, Durham, NC; 5 Edmond and Lily Safra International Institute of Neuroscience of Natal, Natal, Brazil

## Abstract

Several groups have developed brain-machine-interfaces (BMIs) that allow primates to use cortical activity to control artificial limbs. Yet, it remains unknown whether cortical ensembles could represent the kinematics of whole-body navigation and be used to operate a BMI that moves a wheelchair continuously in space. Here we show that rhesus monkeys can learn to navigate a robotic wheelchair, using their cortical activity as the main control signal. Two monkeys were chronically implanted with multichannel microelectrode arrays that allowed wireless recordings from ensembles of premotor and sensorimotor cortical neurons. Initially, while monkeys remained seated in the robotic wheelchair, passive navigation was employed to train a linear decoder to extract 2D wheelchair kinematics from cortical activity. Next, monkeys employed the wireless BMI to translate their cortical activity into the robotic wheelchair’s translational and rotational velocities. Over time, monkeys improved their ability to navigate the wheelchair toward the location of a grape reward. The navigation was enacted by populations of cortical neurons tuned to whole-body displacement. During practice with the apparatus, we also noticed the presence of a cortical representation of the distance to reward location. These results demonstrate that intracranial BMIs could restore whole-body mobility to severely paralyzed patients in the future.

The wheelchair remains the main device to assist navigation in people with motor disabilities, particularly those suffering from severe cases of body paralysis^1^. Up to date, noninvasive BMI approaches, mostly based on electroencephalography (EEG), have been dominant in assistive devices that enable direct brain control over navigation in a powered wheelchair^2^,^3^,^4^,^5^. Although intracranial BMIs hold promise to offer a superior performance over noninvasive systems^6^,^7^ and approximately 70% of paralyzed patients are willing to accept surgically implanted electrodes in their brains to gain control over their assistive devices^8^,^9^, only a few studies have previously attempted to apply intracranial BMIs to wheelchair control^10^,^11^.

One design for an intracranial BMI that controls wheelchair movements is based on joystick movement. This design was previously introduced by our laboratory^10^ and by Xu *et al.*^11^. In our experiments, rhesus monkeys were housed in a home cage equipped with a joystick. They used the joystick to continuously control the movements of an external wheelchair. This control was also performed in BMI mode, where the wheelchair coordinates were extracted from cortical neuronal ensembles^10^. In some BMI experiments the monkeys continued to assist movement with the joystick, in the others the joystick was removed and the monkeys controlled the wheelchair without producing overt arm movements. Recently, Xu *et al.* described how one monkey seated in a wheelchair^11^ was trained to move a joystick in one of four directions to set the direction of wheelchair movement. Under BMI control, this monkey continued to move the joystick, but the control signal was derived from a discrete classifier that extracted joystick position from cortical activity.

These BMI designs are similar to those previously used to reproduce arm movements, only replacing the end actuator with a robotic wheelchair. However, such BMIs cannot be used by severely paralyzed quadriplegic patients, who cannot produce arm movements. Additionally, neural control of arm movements may be an unnecessary intermediary to enable wheelchair navigation. Here we hypothesized that kinematics of whole-body movements could be extracted directly from sensorimotor cortical ensembles, and utilized to control a BMI for wheelchair control. As a first step towards the development of a clinically relevant device of this type, we utilized large scale recordings from multiple cortical areas^7^ obtained by our recently developed multichannel wireless recording system^10^ to enable BMI control over whole-body navigation in a robotic wheelchair.

## Results

**Figure 1:**
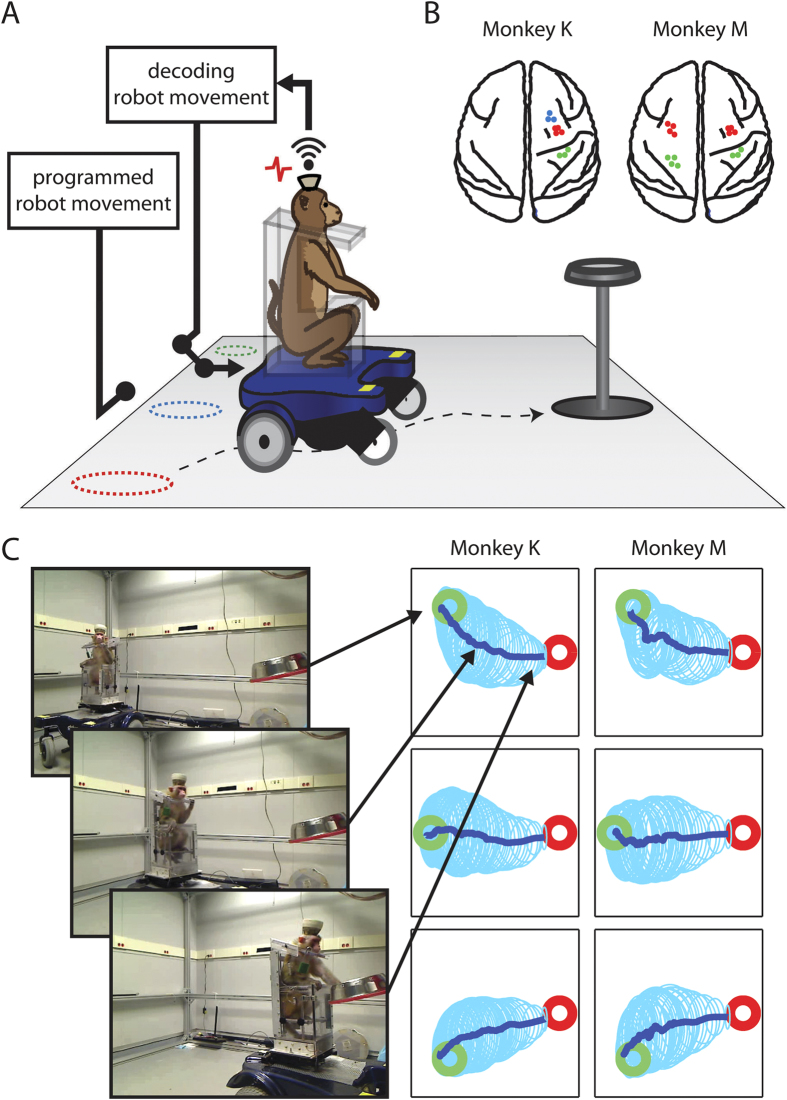
Overview of the experimental design. (**A**) The mobile robotic wheelchair, which seats a monkey, was moved from one of the three starting locations (dashed circles) to a grape dispenser. The wireless recording system records the spiking activities from the monkey’s head stage, and sends the activities to the wireless receiver to decode the wheelchair movement. (**B**) Schematic of the brain regions from which we recorded units tuned to either velocity or steering. Red dots correspond to units in M1, blue from PMd and green from the somatosensory cortex. (**C**) Three video frames show Monkey K drive toward the grape dispenser. The right panel shows the average driving trajectories (dark blue) from the three different starting locations (green circle) to the grape dispenser (red circle). The light blue ellipses are the standard deviation of the trajectories.

The study was conducted in two monkeys (K and M) chronically implanted with multielectrode arrays in multiple cortical areas in both hemispheres (Fig. 1). Neuronal ensemble activity was sampled using our 512-channel wireless recording system^10^. In Monkey K, 79 neurons were recorded bilaterally in the primary motor cortex (M1), 35 in the right primary somatosensory cortex (S1), and 26 in the right dorsal premotor cortex (PMd). In Monkey M, 72 neurons were recorded in bilateral M1, and 72 in bilateral S1.

Monkeys were seated in a mobile robotic wheelchair (Fig. 1). Each trial started with the robot being placed in one of three starting locations. A food reward (grape) was then dispensed onto a plate mounted at the target location, 1.9–2.1 m away. The food location was constant for all trials. Monkeys navigated from the starting location to the food, which completed the trial. At the beginning of each recording session we ran passive navigation trials, with the robot’s routes preprogrammed and identical from day to day (Fig. S1, Movie S1). The passive trajectories were empirically chosen based on two principles. First, we wanted monkeys to perceive that these trajectories, although noisy, clearly headed to the grape dispenser. Accordingly, the training routes were constructed from straight lines toward the grape dispenser, which were distorted by moderate over-turning and correcting-turning. Second, we wanted to enrich the training routes by backwards movements, which monkeys could use to correct navigation errors. Such backward movements were added. Cortical neuronal responses to passive whole-body displacements were utilized to obtain initial settings for two Wiener filters^12^,^13^, which decoded translational (*R* of predictions of 0.62 ± 0.02 and 0.43 ± 0.02 for monkeys K and M, respectively; mean ± standard error) and rotational velocity (0.30 ± 0.02 and 0.22 ± 0.02) from neuronal ensemble activity (Fig. S2, as an example). Note that both translational and rotational velocities were expressed in chair-centered coordinates, not in room coordinates. Accordingly, this control mode was largely independent and unrelated to the locations of objects in the room, such as the food dispenser (see below). After the decoders were trained, the mode of operation was switched to a direct brain-control mode, where the outputs of the Wiener filters operated the wheelchair in real time. As shown below, monkeys were able to learn how to use their brain-derived signals to drive the wheelchair even though the decoding of wheelchair velocity was not superb during the passive navigation training period.

### Neuronal tuning to translational and rotational velocity

**Figure 2:**
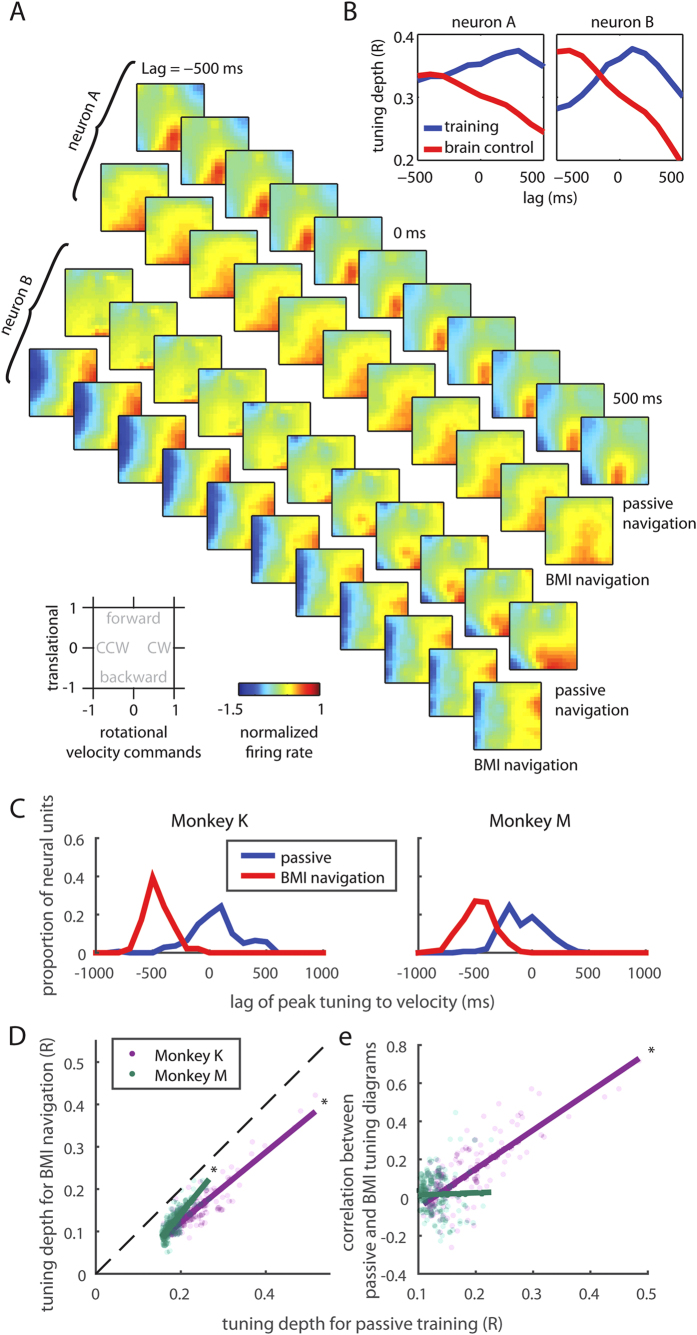
Translational and rotational velocity tuning for neurons during passive training and BMI-based navigation. (**A**) Each diagram shows the normalized firing rate as a function of translational and rotational velocity commands (bottom-left), where 1 represents the maximum command value sent to the wheelchair (positive values represent forward commands for translation, and clock-wise (CW) for rotation). Each sequence shows the normalized firing rate at different time lag. Neuron A is tuned to backward movement during both passive training and BMI control. Neuron B is tuned to right turn movement during brain control, but with different tuning during passive training. (**B**) The tuning depth across time for the two neurons in (**A**). Note that the time lag of the peak tuning differs between passive training and BMI control. (**C**) Distribution of the tuning depth to velocity and the lag of peak velocity tuning for all the neural units recorded. The lag of peak velocity tuning moved from ~0 ms to ~ −500 ms, i.e. half a second earlier than the movement onset. (**D**) Neural units that were well tuned during passive navigation, were more likely to be well tuned during BMI navigation. Each dot represents one neural unit, and the solid line is the regression line. (**E**) For units that were better tuned, their tuning diagrams between passive and MI navigation were also better correlated in Monkey K, but not in Monkey M.

Individual neurons were tuned to the wheelchair movements during both passive and BMI navigation (*Tuning Depth* of 0.24 ± 0.005 and 0.18 ± 0.002 for passive movements, for monkeys K and M, respectively; and 0.15 ± 0.005 and 0.12 ± 0.003 for BMI control; see Supplementary Methods). Color plots of Fig. 2A show tuning patterns to translational and rotational velocity for two representative neurons (neurons A and B), both recorded in Monkey K’s M1. Again, these plots refer to wheelchair-centered coordinates, not to room coordinates. During passive navigation, neuron A increased its firing rate when the robot moved backward, whereas neuron B increased its firing rate when the robot moved backward and/or rotated clockwise. During BMI-based navigation, neuron A remained tuned to backward movement, whereas neuron B was no longer tuned to backward movements, but remained tuned to clockwise rotations.

### Tuning depth analysis

Following an approach previously introduced by us to analyze the Wiener filter performance in BMI control^14^, we calculated tuning depth as a function of time lag between neuronal activity and robot movement for the entire sample of recorded neurons in the two monkeys (Fig. 2B). This analysis revealed that tuning depth reached its maximum before wheelchair movement during BMI control versus during passive navigation. This result was observed for the entire neuronal population recorded in the two monkeys (Fig. 2C, Wilcoxon signed rank test, p < 0.01 for both monkeys) and for M1 and S1 populations analyzed separately (Wilcoxon signed rank test, p < 0.01 for both monkeys and both regions). This finding likely indicates the difference in causal relationships between the two modes of operation: during passive navigation, neuronal responses were caused by robot movements, whereas during BMI navigation, robot movements were caused by neuronal modulations that preceded the actual robotic wheel chair movement (Fig. S3). Additionally, maximum tuning depth occurred earlier for M1 neurons than for S1 neurons during BMI control. This time difference was statistically significant for Monkey K (−489.5 ms in M1 vs −431.6 ms in S1; Wilcoxon Rank Sum Test, p < 0.01) and marginally missed the 0.05 significance criterion for Monkey M (−495.7 ms vs −441.3 ms; p = 0.055). During passive movements, the time difference was not significant (p > 0.314). This finding supports previous studies that demonstrated a lead of M1 activity compared to S1 during voluntary movements^15^. Notice, however, that in the current study movements were initiated through a BMI without the overt production of body movements.

Consistent with our previous studies where we compared neuronal tuning characteristics between the period when a decoder was trained and the period during BMI operations^14^,^16^, the characteristics of neuronal tuning to the wheelchair movements differed between passive navigation and brain control in two ways. First, we observed reduction in tuning depth during BMI navigation. However, neurons that were better tuned during passive navigation were also better tuned during BMI operations, as the tuning depth was actually positively correlated when passive navigation was compared with brain control across all neurons (Fig. 2D. Mann-Kendall Test, p < 0.01). Second, tuning diagrams could change substantially for many neurons after the BMI control started (Fig. 2E & Fig. S4). In particular, there was a considerable mismatch between the tuning diagrams for neurons that were weakly tuned. As shown in Fig. 2E, neurons that were less tuned to velocity commands had lower correlation between BMI and passive navigation in their tuning diagrams (calculated as pixel-to-pixel correlation). This mismatch was particularly strong for Monkey M, where the mismatch was also clear for the well-tuned neurons (Mann-Kendall Test, p = 0.126). The presence of such a mismatch in weakly tuned neurons could reflect the fact that tuning characteristics during BMI control were particularly dependent on the overall synchrony in the neuronal ensemble, the property that changes during BMI learning^16^. This interesting finding will be investigated further in a future study. In Monkey K, well-tuned neurons tended to retain their tuning properties after the transition to BMI control (Mann-Kendall Test, p < 0.01).

### Behavioral improvement over time

**Figure 3:**
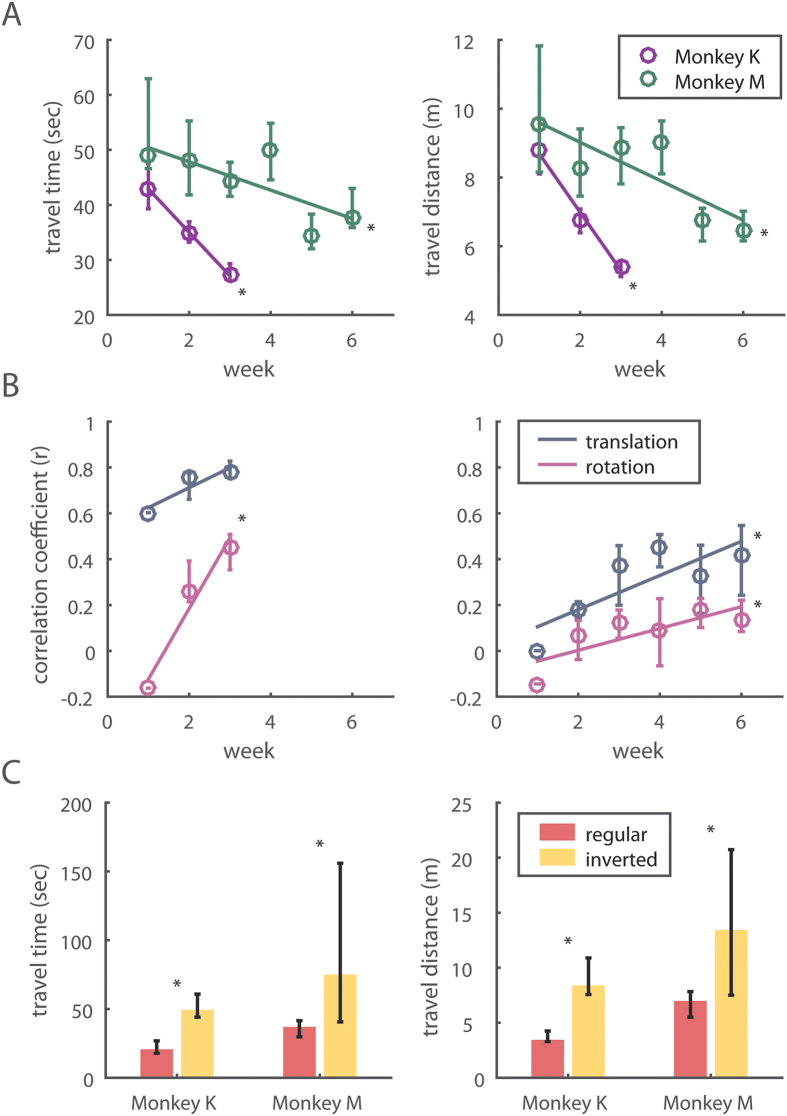
Behavioral improvement and the increase of decoder similarities across sessions. (**A**) Both monkeys show significant improvement in the traveling time and distance as they learn. The circles represent the median and the error bars show the interquartile range of the medians. (**B**) Increased correlations between decoders trained in earlier sessions and the last session. (left, Monkey K; right, Monkey M (**C**) Both monkeys demonstrate impaired performance once their decoded movement commands were inverted (forward now becomes backward, and right turn becomes left turn). The bar graph shows the median and the error bars indicate the interquartile range of the medians.

As the training continued for 3 weeks for Monkey M and 6 weeks for Monkey K, BMI navigation gradually improved for both monkeys, as evident from the reduction in trial duration (Fig. 3A, Mann-Kendall Test, p = 0.025 and 0.045 for Monkey K and M) and trajectory length (Fig. 3B, Mann-Kendall Test, p = 0.008 and 0.020 or Monkey K and M). Monkey K started with a 43.1 s trial duration (median value for the first week), and improved it to 27.3 s (last week). Monkey M improved from 49.1 to 34.7 s. These performance improvements were accompanied by subtle changes in neuronal tuning. We observed that the decoder settings gradually changed from session to session. Taking the last recording session as a reference, we observed that the decoder settings were very distinct from those in the earlier recording sessions (Fig. 3B, Mann-Kendal Test, Monkey K: p = 0.256 and 0.002 for translational and rotational velocity commands; Monkey M: p = 0.032 and p = 0.042 for the two commands). This suggests a convergence to a neuronal tuning pattern, which was optimal for brain control.

### Inverting decoders decreased performance

After both monkeys reached their best performance levels, we tested whether this improvement resulted from directionally tuned neuronal activity or from non-directional factors (e.g. temporal patterns of neuronal modulations). To change the BMI directional output without changing non-directional components, we reversed the sign of both translational and rotational velocities (i.e., forward instead of backward movement and counterclockwise rotation instead of clockwise Fig. S5). After this operation, navigation accuracy decreased significantly for the two monkeys (Fig. 3C, Wilcoxon signed rank test, p < 0.01 in both trial duration and trajectory length for both monkeys). This observation confirmed that monkeys indeed learned to use their directionally tuned cortical neurons to navigate the robotic wheelchair and a random walk strategy did not yield the desired movement toward the reward location.

### Monkey head orientation

The video tracking of monkey head position showed two basic head orientations: (1) toward the food reward, and (2) straight relative to the chair. The first orientation was evident from the distribution of monkey head orientation relative to the feeder as the function of trial time (Fig. S6). While the monkeys turned their heads in different directions, they looked at the grape feeder more often than at different room locations. This tendency was especially prominent in Monkey K who clearly looked at the feeder throughout the trial. In both monkeys, head orientation at the feeder became especially prominent when the wheelchair arrived at the feeder location. The second type of head orientation, i.e. straight relative to the chair was clear from the distribution of head angle relative to the chair (Fig. S7). The tendency to look straight was especially strong in Monkey M and less strong in Monkey K who, as shown in the previous analysis, often looked at the food location.

These basic head orientations were also evident from two correlation analyses. In the first analysis, the angle of wheelchair orientation was negatively correlated with the head angle relative to the chair (r = −0.50, p < 0.01 for Monkey K and r = −0.19, p < 0.01 for Monkey M; angular-angular correlation analysis). In other words, monkeys turned their heads in the direction opposite to the chair rotation when the chair turned away from the food dispenser. In the second analysis, the angle of wheelchair orientation was positively correlated with the head angle in room coordinates (r = 0.61, p < 0.01 for Monkey K and r = 0.83, p < 0.01 for Monkey M; angular-angular correlation analysis). This corresponded to the monkeys looking straight relative to the chair.

### Neuronal representation of distance of the wheelchair to the reward location

**Figure 4:**
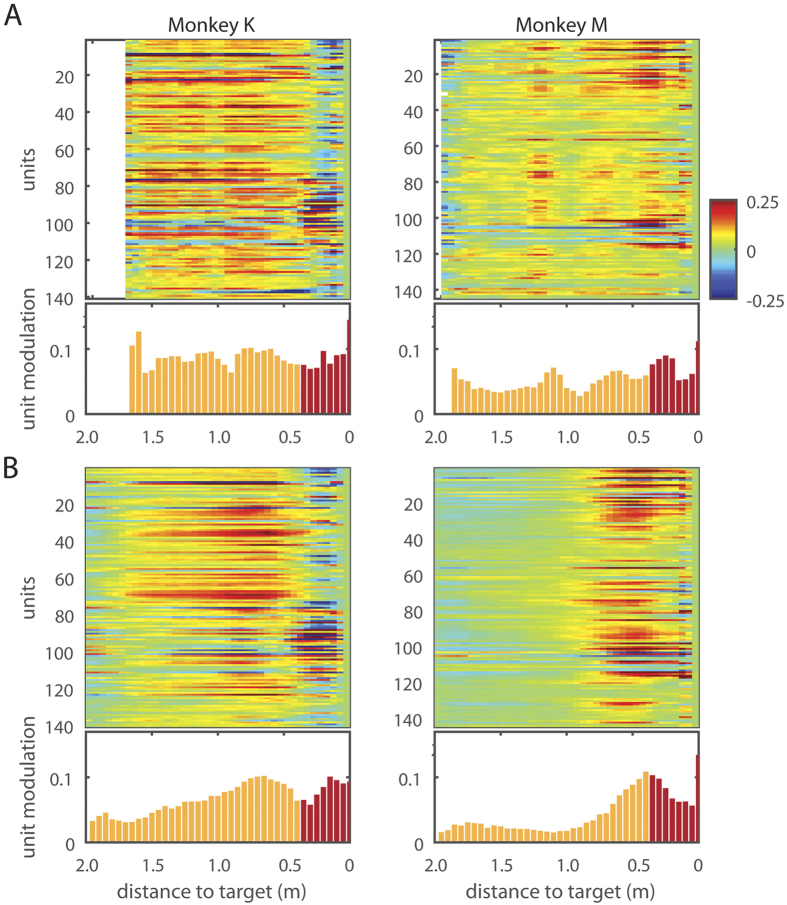
Population responses as a function of distance to the reward. (**A**) Population responses in Monkey K and M as a function of distance to the reward during passive navigation and BMI navigation (**B**) Color represents normalized firing rate, and the bar graph shows the average unit modulation, calculated by the average of the absolute values of all units’ normalized firing rate at each distance. When the cart is getting close to the target, the monkeys may reach to the grape, and these reach-related activities are shaded in red.

Although BMI decoding did not incorporate absolute position of the robot as a controlled parameter and relied on the robot-centered coordinates instead, we observed the presence of positional tuning to reward location in cortical neurons during the BMI navigation task. This positional tuning is depicted by the peri-distance histograms (PDHs) that represent neuronal activity as a function of the distance of the wheelchair to the food dispenser (Fig. 4). The positional tuning could not be explained by the monkey arm movements because it occurred before the wheelchair reached the docking range, and the monkeys did not move their arms during this period. The positional dependence in the population PDHs was specific to the periods of BMI control mode in both monkeys (Fig. 4B, Mann-Kendall trend test, p < 0.01 for both monkeys). No such dependence was observed during passive navigation (Fig. 4A). In Monkey K, neuronal firing rates gradually increased as the robot approached the feeder (Fig. 4). In Monkey M, there was no such increase until the robot was 0.9 m from the feeder. At this spatial position, neuronal firing rates started to increase as the robot approached the feeder (Fig. 4). Although we found the presence of this tuning in brain-control trials, we did not detect the exact instance when this tuning emerged during the training period.

Offline Wiener filter decoders were then trained to predict distance to target from cortical ensemble activity for both monkeys. Consistent with the PDHs of Fig. 4, this analysis predicted distance to the reward location from neuronal population activity. R of predictions during brain-control were 0.49 ± 0.02 and 0.31 ± 0.01 for Monkey K and Monkey M, respectively. Curiously, while the PDHs for passive navigation mode did not show a steady rate of increase, the Wiener filters trained from data within this model also predicted distance to reward (Fig. 4). This is because these PDHs still contained neuronal modulations, which the Wiener filter picked and utilized to generate predictions. R of predictions (mean ± standard error) were 0.42 ± 0.02 and 0.22 ± 0.02 for Monkey K and Monkey M, respectively. However, the filters trained specifically to decode distance for either mode do not generalize to the other. Specifically, R of predictions for brain-control mode with filters trained in passive mode were 0.05 + /−0.09 and 0.08 + /−0.04; R of predictions for passive mode with filters trained in brain-control were 0.21 + /−0.12 and 0.24 + /−0.09, for Monkey K and Monkey M, respectively. This observation indicates that the distance tuning cannot be attributed to reward related modulation alone. Moreover, cortical neuronal tuning to target distance cannot be explained by the monkeys’ overt reaching movements, since the animals did not produce arm movements until the robot arrived at the food dispenser location (Movie S1). Arm reaching for grapes did produce neuronal modulations (highlighted in red in Fig. 4), but the comparison of neuronal modulation magnitudes during arm movements with those during navigation showed only a weak correlation on a neuron to neuron basis (Fig. S8). This indicates that the neuronal firing modulations observed during wheelchair navigation could not be explained as being related primarily to the production of arm movements.

## Discussion

The present study has demonstrated that intracranial wireless cortical ensemble recordings can be harnessed to control whole-body navigation in a mobile device such as a robotic wheelchair. Heretofore, intracranial BMI research in primates has focused primarily in reconstructing isolated upper limb movements^12^. A few years ago, our laboratory introduced the use of BMIs to investigate the possibility of restoring more complex movements, such as bipedal locomotion^17^ and then bimanual movements^16^. Yet, the investigation of cortical representation of whole-body translations, and the likelihood of using intracranial BMIs for wheelchair control, has been almost completely neglected. A few primate studies on spatial representation of the environment by hippocampal neurons^18^,^19^,^20^ have been performed, but these results are not directly applicable to the BMI for wheelchair control. Neuronal mechanisms of spatial encoding in rodents have received much more attention^21^,^22^,^23^ compared to primate studies, but the prospects of BMI navigation based on these neuronal properties was not explored.

Schwartz *et al.*^10^ demonstrated a joystick-based BMI paradigm where monkeys controlled movements of a wheelchair by their cortical modulations. The monkeys were not seated in the wheelchair in this study. More recently, Xu *et al.* trained one monkey to steer while seated in a wheelchair using a hand-held joystick to generate discrete but not continuous navigation commands^11^. These authors also demonstrated a BMI version of this joystick control, where hand movements were decoded from M1 ensemble activity to produce steering commands^11^. This group, however, did not attempt to translate cortical activity directly into whole-body navigation, without using hand movements as an intermediary. In another study, monkeys navigated in a virtual environment using a joystick while their bodies remained stationary^24^. Under these conditions, neuronal firing modulations observed in the medial superior temporal cortex depended on whether monkeys actively steered with the joystick or passively observed the visual flow of the scene.

In contrast to these studies, we trained our BMI decoder using a passive wheelchair navigation paradigm. A somewhat similar approach was explored in several BMI studies, where subjects passively observed the movements of an external device and/or imagined voluntarily controlling those movements while the BMI decoder was trained^16^,^25^,^26^. This BMI training approach has obvious clinical significance since severely paralyzed subjects cannot produce overt body movements to train a BMI decoder. We observed that, after passive navigation was employed to set up the decoder parameters, animals significantly improved their navigation performance through learning, likely mediated by widespread cortical plasticity^16^.

It is important to emphasize that, different from previous BMI studies in which animals were trained by simply observing movements of a virtual actuator on a computer screen (e.g. virtual arm, legs, etc.), here the entire animal’s body was passively transported by the actuator (wheelchair) during training of the BMI decoder. This implies that an integration of vestibular, proprioceptive, visual and auditory inputs influenced the choice of our optimal decoder settings, something that had not been tried before in the BMI field. Given the nature of our task, it is very likely that multimodal sensory inputs continued to influence neuronal ensemble activity during BMI control. In this study, we chose the training routes empirically. In the future, it would be of interest to optimize these routes to maximize decoding accuracy. Optimizing the decoder is equivalent to maximizing the signal-to-noise ratio (SNR). The SNR could be increased by searching for the wheelchair movement patterns, which elicit stronger and more consistent neuronal responses, for example optimal ranges of wheelchair velocities and accelerations. In addition to optimizing the passive training procedure, it would be of interest in the future to expand the recording areas, so that neural information is recorded from cortical and subcortical areas that represent relevant visual and vestibular inputs. Since we recorded mostly from M1 and S1, it is possible that vestibular and visual signals were underrepresented, and better BMI control could be achieved if we sampled from associative areas of parietal and frontal cortices.

We observed distinct differences in neuronal firing patterns during passive navigation and when monkeys were switched to the BMI control phase of our experiments. Most notably, cortical neuronal tuning peaked prior to the wheelchair movement during BMI control, likely indicating a causal relationship between cortical activity and the type of navigation pattern generated by the animal. During passive navigation, neuronal tuning peaked after the wheelchair moved because the cortical neuronal activity was mainly induced by feedback sensory signals generated by the passive animal body movements. An approximately 500 ms delay between neuronal modulations and the wheelchair response during BMI control is not surprising because there was a mechanical delay to translate the velocity commands to the wheelchair movement. In the future, optimization strategies for the decoder should be explored to account for this delay. In the present study, the 500 ms lag was right in the middle of the 1000 ms time window used for decoding of neural data, which assured that neural data was collected from the most informative epoch. However, some fine adjustments could be helpful in the future, particularly those based on adaptive decoders. This heterogeneity in the maximum tuning time concurs with our previous results for BMIs aimed at enacting arm^14^ and leg^17^ movements. In addition to these time lag-dependent changes, learning the BMI control evoked changes in directional tuning of cortical neurons to translational and rotational velocity of the wheelchair. Based on these findings, we suggest that both temporal and directional characteristics of neuronal tuning adapted to improve BMI control. This conclusion is in line with similar findings for upper-limb BMIs^12^,^27^.

We also observed the emergence of a cortical representation of distance to the reward, which was specific to the context of the task chosen to test our whole-body BMI. Remarkably, the emergence of distance tuning in cortical neurons was not related to the kinematic variable controlled through the BMI (wheelchair linear and rotational velocity encoded in the chair-centered coordinate system). Instead, this additional cortical representation apparently emerged during training and become superimposed to the neuronal velocity mapping already present in the sensorimotor cortex. The representation of the distance to reward, which also served as a target for the wheel chair movement, is likely to be somewhat related to reward encoding by cortical neurons, which was recently demonstrated by Marsh *et al.*^28^ using an arm-reaching task. Indeed, an increase in neuronal activity with the proximity to the reward could indicate a reward-anticipation signal. However, PDHs were very different during passive navigation trials and BMI control, despite the fact that reward was present in both cases (Fig. 4). In the former case, PDHs were near-flat and in the latter they showed a steady increase in activity. This finding points to factors different from a simple reward expectation signal as the main contributor to distance tuning. Likely, the steady increase in neuronal activity with target proximity reflected an increase in the level of animal engagement in the brain-control task. Indeed, when the monkeys actively controlled the wheel chair steering using their brain activity they probably attended more to the final target than when they were passively moved towards the end goal since in the latter condition the monkeys’ reward was guaranteed. Numerous previous studies offer possible contributing factors to the emergence of such a distance to reward tuning. For instance, cortical encoding of spatial attention, independent of the representation of motor goal and working memory, could have played a key role in our paradigm^29^,^30^,^31^. Furthermore, anticipatory buildup in cortical neuronal activity has been demonstrated by many researchers^14^,^31^,^32^,^33^ as potentially representing motor preparation (preparation of arm reaching in our case), spatial encoding^30^, and mirror-like representations^34^,^35^,^36^ where cortical activity reflects an observation of movement. All these potential factors, in principle, can be independently tested to quantify their individual contribution to the distance to target tuning observed here. However such manipulations were beyond the scope of the present study and will be left for examination in future experiments. Moreover, we also intend to incorporate wireless multichannel, surface EMG recordings, to this setup to quantify whether any residual arm movements could contribute to these neuronal firing modulations.

Irrespective of its origin, we learned that the distance to reward could be decoded offline using the cortical neuronal ensemble activity recorded in our experiments, yielding very reasonable and accurate predictions. Previously, we had decoded target location for an arm reaching task, where target location was also not part of the BMI control variables^37^. The presence of such novel cortical representation suggests that, in the future, potentially new control parameters may be extracted from cortical neuronal ensembles, in addition to the variables normally employed to control a BMI. Indeed, such emergent properties could add a new level of versatility to BMIs. Recently Marsh *et al.*^28^ proposed that cortical representation of reward should be incorporated in BMI design to make it more autonomous. Our study adds to these results by describing the existence of neuronal signals which modulate not just the presence or absence of reward, but a continuous representation of reward location; a tuning of space that coexisted with the representation of chair velocity. In this context, future studies should explore the possibility that cortical neuronal ensembles engaged in navigation could trigger an incorporation of the navigated space (not necessarily related with reward location only) into novel cortical representations.

Earlier we argued on a similar ground that prolonged BMI use leads to the assimilation of the artificial tool, e.g. robotic or virtual arms/legs, into the body representations found in the subject’s cortex^35^. Some evidence of such integration has been reported already for manually controlled wheelchairs^38^,^39^,^40^,^41^. Our current observations of neuronal tuning to wheelchair kinematics and target distance after the transition to BMI control adds to these findings and suggests new directions for future scientific inquiries on how prolonged BMI operation affects cortical representation of the sense of self and the peri-personal space.

Overall, the whole-body navigational BMI introduced here constitutes the natural evolution of our lab’s experimental approach of employing large-scale intracranial neuronal ensemble recordings as the main control signal source for neuroprosthetic devices^6^,^10^. Although we have seen a surge in the use of such BMIs in humans^25^,^26^,^42^, the majority of current clinical BMI applications are still dominated by noninvasive rather than intracranial BMIs. Part of the reason for this preference likely comes from the fact that intracranial BMIs have produced mixed clinical results, including an implant malfunction and failure, and motor outcomes that are not much superior to those obtained by non-invasive technology^43^,^44^,^45^. A key issue accounting for these lackluster clinical results is related to the deterioration of neural recordings produced with Utah probes^26^. Recently better results were reported for one human patient from whom ~200 motor cortical neurons were recorded for 13 weeks^25^, but it remains to be seen how consistent these implants are. On the contrary, chronic implants in nonhuman primates based on flexible microwire arrays or cubes have been shown to yield good quality recordings for more than 5 years, even when hundreds of these metal filaments are implanted in the cortex^10^. In this context, microwire arrays maybe a better option than Utah probes for human chronic implants. Yet, in our judgment, more animal experimentation will be needed before reliable intracranial implants can be offered for humans^6^.

In the wheelchair domain, numerous noninvasive EEG-based BMIs have been described. These applications employed motor imagery^46^ and P300 potentials^47^ as the source of brain-derived motor commands. Although these systems perform with an acceptable (80%) success rate in tasks that involve predefined paths and target locations, and can be improved to cope with real environments^48^, they are clearly limited and cannot be considered as the final solution for routine clinical use in the future. One approach to improve these systems is to apply shared control schemes where some commands are delegated to the robotic system^49^,^50^. With regard to our current study we plan to integrate navigation in complex environments in future experiments.

The potential of intracranial BMIs for whole-body navigation, as demonstrated in our study, lies on the generation of continuous kinematic control signals, which are needed for subjects to navigate through complex and unknown spaces. Since typical EEG based wheelchair design only supports discrete control, it is clearly insufficient to generate continuous trajectories that can change at a moment’s notice. Yet, because of their low risk, EEG based systems will probably remain the dominant clinical BMI approach for a while, and even expand to include new applications, like, EEG controlled exoskeletons. However, as intracranial recording systems improve in efficiency and safety, they will likely become more attractive to the clinicians and patients, particularly those suffering from devastating levels of body paralysis, in the future.

In conclusion, the current results promise much more than a mere demonstration that monkeys can control whole body navigation. Indeed, the finding that cortical ensembles can adapt to a whole-body navigation task propels BMIs to a new dimension and creates innovative avenues for exploration of this approach’s clinical relevance in the future. In this context, the present results support our recent clinical observation that when paraplegic patients are subjected to intense BMI training, they not only became capable of regaining walking, using a robotic exoskeleton, but they also can exhibit signs of partial neurological recovery of sensorimotor and autonomic functions^51^. Based on our experimental and clinical observations, we raise the hypothesis that BMIs can lead to partial neurological recovery or even augment brain function because their chronic and continuous use may trigger widespread cortical plasticity and the emergence of new cortical representations. As such, BMIs will likely have a profound clinical impact in the future.

## Materials and Methods

### Study Design

The objective of this study was to demonstrate whole-body navigation with control signals derived from neuronal ensemble recordings in multiple cortical areas. Notably, our design did not require the subjects to map overt movements to the navigational direction, which makes this paradigm applicable to the needs of severely paralyzed patients who cannot move their limbs but desire to restore whole-body mobility.

Two adult rhesus macaques (Monkey K and M) were used for the study. All animal procedures were performed in accordance with the National Research Council’s Guide for the Care and Use of Laboratory Animals and were approved by the Duke University Institutional Animal Care and Use Committee. The two monkeys were chronically implanted with arrays of microwires in multiple cortical areas of both hemispheres (M1, S1, and PMd). Our recently developed multi-channel wireless recording system was employed to sample from hundreds of neurons in sensorimotor cortex simultaneously^10^. Both monkeys learned to navigate in a room while seated in a mobile robotic wheelchair using their cortical activity as the navigation control signal. Cortical ensemble recordings were converted to steering commands for the mobile robotic wheelchair based on linear decoding algorithms.

Both monkeys successfully acquired the ability to steer the robotic wheelchair towards a grape reward. They achieved two dimensional navigation with multiple starting positions and orientations of the robot.

### Task Design

The monkeys were operantly conditioned to drive the mobile robotic wheelchair toward a food reward (grape). The rewards were delivered by an automated grape dispenser. Each experimental session consisted of about 150 trials during which the monkeys navigated to the grape from a starting location, reached for the grape and placed it in the mouth. The robotic wheelchair was then driven away from the grape dispenser to a new starting location.

The first 30 trials were used to train BMI decoders. During these training trials, the monkeys had no control over the movements of the robotic wheelchair. Rather, the experimental control system navigated the wheelchair along several predetermined routes toward the grape dispenser (Fig. S1). These training routes were chosen empirically. They represented distorted straight lines toward the grape dispenser. The distortions were achieved by adding moderate turns and backward movements. The data obtained during this passive navigation was used to train L2-norm Wiener Filters, which generated steering commands from a 1 s window divided into ten 100 ms bins to count spikes produced by each neuron in the ensemble. The filtering outputs were calculated as sums of the spike counts multiplied by the filter weights.

Once the decoders were trained, we proceeded to BMI trials during which the robotic wheelchair was steered directly by the cortical signals. The monkeys would navigate themselves toward the grape, while the wheelchair returning to a starting location was the only part performed automatically by the program. (Obviously the monkeys did not want to move away from the food location.)

In the easiest version of the task, the two monkeys performed 1D navigation (8 sessions for Monkey K; 9 sessions for Monkey M). Here, the monkeys controlled only the forward and backward movements of the robotic wheelchair. The main, more challenging task required the monkeys to navigate in 2D. Their cortical activity was converted into both translational (backward or forward) and rotational (leftward or rightward) velocity components.

The translational velocity was limited to −0.28 to 0.28 m/s (negative values for backward movements and positive for forward), and rotational velocity was limited to −46 to 46 degrees/s (negative for leftward, positive for rightward). If the decoded velocity commands exceeded the limit, the command sent to the robotic wheelchair would be set to the limit value. In the 2D task, the robotic wheelchair could be initially placed at one of the 3 starting locations, whose coordinates were (0.62, 0.50), (0.88, 0), and (0.62, −0.50) meter, where (0, 0) was the room center.

The monkeys navigated in a 3.5-by−2.5 m experimental room. The actual drivable area was chosen to be 3.1-by−2.4 m, which assured safety. When the robotic wheelchair was at the drivable area boundary, the program would stop the robotic wheelchair if the decoded commands would have moved the robot through the boundary, and would only execute the decoded commands if the robot moved inside the drivable area. Additionally, when the robotic wheelchair got close to the grape dispenser (docking range), the program would take over the control and park the robot automatically to ensure the monkeys could comfortably obtain their rewards.

### Electrode Implantation

Monkey M was implanted with four multielectrode arrays. Within each array, Isonel coated, stainless steel polyimide insulated microelectrodes were grouped in two four-by-four, uniformly spaced grids each consisting of 16 microwire triplets of different lengths, for a total of 96 microelectrodes. Although the implants were placed bilaterally in the primary motor (M1) and primary somatosensory cortices (S1) in both monkeys, in this study we recorded only from the right-hemispheric implants in Monkey K due to their superior neuronal yield. Each hemisphere received two arrays: one for the upper and one for the lower-limb representations (for a total of 384 microelectrodes, Fig. 1). In the current experiment we used 256 channels in Monkey M to record from neurons in the bilateral arm and leg areas of M1 and S1. Monkey K was implanted bilaterally with six multielectrode arrays containing 96 microwires each. Stainless steel microwires 30–50 μm in diameter were grouped into bundles of three with a single leading conical microwire surrounded by two other microwires with cut angled tips^10^. The arrays were implanted in the arm and leg representation areas of M1 and S1 and also in the bilateral premotor cortices totaling 576 channels. For the current study we recorded from 128 channels in Monkey K which were from bilateral M1 and premotor cortex and right side S1. Extracellular neuronal recordings were obtained using an in-house built wireless recording system described previously^10^.

### BMI Navigation System Design

The BMI navigation system had three main components: *(1) experiment control system*, *(2) wireless recording system*, and *(3) robotic wheelchair* (Fig. S9). The experiment control system controlled the experimental sequence, performed BMI decoding of neuronal ensemble activity, and handled the video tracking of both wheelchair and monkey head pose. The wireless recording system recorded neuronal ensemble activity from the monkey brain and sent the neuronal data to the experiment control system. The robotic wheelchair accommodated the monkey chair and received driving commands wirelessly from the experiment control system. The three components communicated with each other in a local network.

### Experiment Control System

The experiment control system controlled the flow of the experiment, which included controlling the experimental sequence, running BMI decoding algorithms that computed navigation commands for the robotic wheelchair, controlling the robotic wheelchair, and video tracking the wheelchair and the monkey head pose.

The location and orientation of the robotic wheelchair was tracked using ASUS Xtion camera (640 × 480 pixels, 30 frames/sec) mounted on the ceiling of the experiment room. The video tracking software was written in C + + and built with OpenCV library. The software processed the video stream, and segmented the video frames to determine the robotic wheelchair location. The wheelchair orientation was determined based on the markers located at the front and the back of the robot. The robot position signals were smoothed using a Kalman filter. Similarly, we put color markers on the cover of the monkey headcap to track its location and orientation the same way as we tracked the wheelchair.

### Statistical Analysis

We ran Monkey K for 21 sessions and Monkey M for 28 sessions, and sometimes two sessions in a day. Two sessions of Monkey K and five sessions of Monkey M were excluded from the analysis because (1) the sessions had less than 10 BMI navigation trials (3 sessions), (2) technical issues occurred during recording (1 session), (3) a decoder was ill-trained and biased toward negative velocity (1 session), or (4) communication between the experiment control system and the Raspberry Pi on the robot failed frequently (2 sessions). On average, each session yielded 49.0 ± 32.0 and 44.4 ± 9.3 BMI navigation trials by Monkey K and M. In the end, 19 sessions from Monkey K and 23 sessions from Monkey M entered the analysis below.

### Tuning Diagram

To characterize tuning diagrams for each neural unit at different time lags (e.g., Fig. 2A), we first aligned the controlling commands (i.e., translational and rotational velocity commands) of the robotic wheelchair to the neural activities (spike counts of 100 ms time bin). Next, the spike counts were normalized by subtracting the mean and dividing by the standard deviation of the spike counts of this unit. The range of the controlling commands were both divided into 18 bins, which formed the two axes of the tuning diagram (18 × 18 bins). Then, we sorted the normalized spike counts into the diagram based on the corresponding controlling commands, and averaged the normalized spike counts that fell into the same bin. Lastly, missing values of the tuning diagram were filled in and smoothed by the mean of neighbors in both space and time. To quantify how similar two tuning diagrams were, we calculated Pearson correlation between the two diagrams.

### Neuronal Modulation to Distance to Target

The firing rates for each session were first binned into 10 ms-bins and normalized against the session averages. The maximum range of distance the wheelchair traveled to was divided into 20 bins. For each session, the normalized firing rates of all neurons corresponding to when the monkey was within a particular distance bin during each regime (training vs. brain-control) were averaged to obtain the session average. The resulting session averages were then averaged to obtain the total average. These were plotted in Fig. 4’s raster plots.

To obtain the histograms from the total session averages, we took the average of the absolute values of all neuron’s normalized firing rates at each distance bin. The reach-dependent distance (the bars colored red in Fig. 4) was a conservative estimate of when a monkey may reach the grape dispenser.

## Additional Information

**How to cite this article**: Rajangam, S. *et al.* Wireless Cortical Brain-Machine Interface for Whole-Body Navigation in Primates. *Sci. Rep.* **6**, 22170; doi: 10.1038/srep22170 (2016).

## Supplementary Material

Supplementary Information

Supplementary Movie 1
