# Supplementary material for: Wireless Cortical Brain-Machine Interface for Whole-Body Navigation in Primates
Source: Sci Rep. 2016 Mar 3;6:22170. doi: 10.1038/srep22170 (PMC4776675; doi:10.1038/srep22170)
Supplement: Supplementary Information [file srep22170-s1.pdf]

Supplementary Materials

Wireless Cortical Brain-Machine Interface for Whole-Body Navigation in Primates

Sankaranarayani Rajangam[1,2†], Po-He Tseng[1,2†], Allen Yin[2,3], Gary Lehew[1,2], David Schwarz[1,2],

Mikhail A. Lebedev[1,2], Miguel A. L. Nicolelis[1,2,3,4,5]*

[1] Department of Neurobiology, Duke University Medical Center, Durham, NC.
[2] Duke Center for Neuroengineering, Duke University, Durham, NC.
[3] Department of Biomedical Engineering, Duke University, Durham, NC.
[4] Department of Psychology and Neuroscience, Duke University, Durham, NC
[5] Edmond and Lily Safra International Institute of Neuroscience of Natal, Natal, Brazil.

Supplementary Figures and Legends

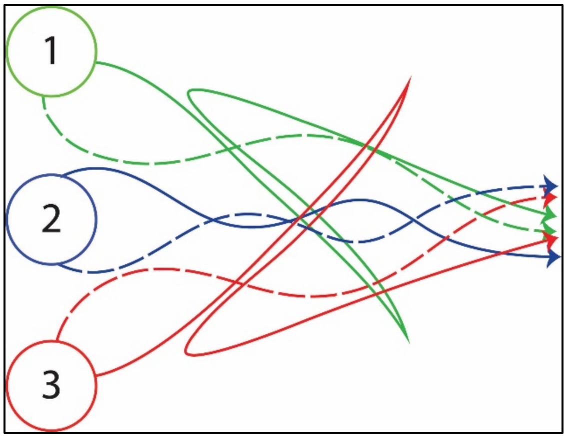

Figure S1. Schematic of training routes. The 6 training routes from three starting locations to the grape dispenser. The circles represent the car starting location in the room. The arrows denote the location of the grape dispenser. The bold and dashed lines represent two routes per starting location.

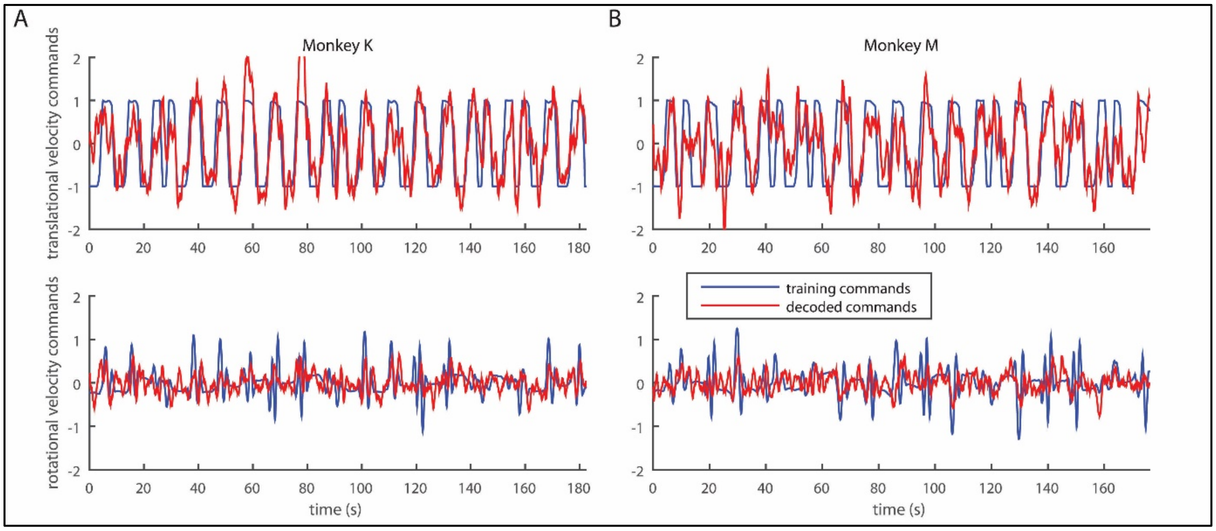

Figure S2. An example of decoded translational and rotational velocity commands during passive navigation. (A) For Monkey K (R = 0.71 and 0.40 for translational and rotational velocity commands) and (B) Monkey M (R = 0.53 and 0.35 for translational and rotational velocity commands). Prediction was evaluated by 5-fold cross validation.

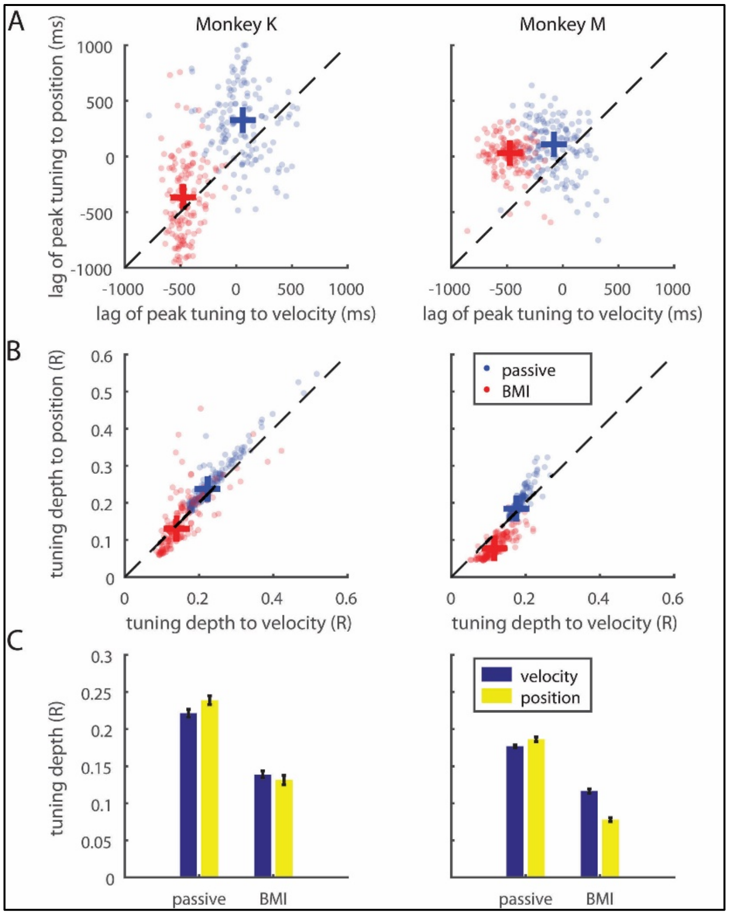

Figure S3. Tuning depth analysis. (A) The timing of peak tuning in both velocity (translation and rotation) and position (2D plane) were shifted earlier during BMI navigation than in passive navigation (Wilcoxon signed rank test, $p<0.01$ for both monkeys). Each dot represents one neuron under a navigation mode, and the cross indicates the median of all neurons. (B) The tuning depth (R) decreased when shifted from passive navigation to BMI navigation (Wilcoxon signed rank test, $p<0.01$ for both monkeys). This result was expected as the decoder used during BMI navigation was trained during passive navigation. The crosses indicate the median of all neurons, and the medians are also plotted in (C). The error bars represent the upper and lower quartile of the median. While the neurons were better tuned to position during the passive training, they were better tuned to velocity during brain control (ANOVA, interaction $p=0.0675$ for Monkey K, and $p<0.01$ for Monkey M).

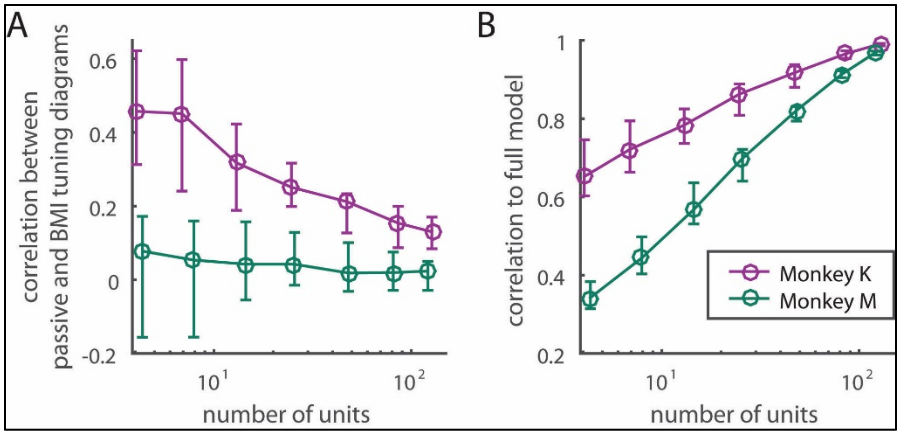

Figure S4. Better tuned neurons had more consistent tuning diagrams for Monkey K. Supplement to Fig. 2E, which shows that a neuron's tuning diagrams between passive and BMI navigation were better correlated if the neuron is better tuned for Monkey K, but not for Monkey M. This figure shows the same result, but with a group of neurons that were selected by L1-regularized regression (lasso). Lasso selected a set of neurons that best decode the translational and rotational velocity commands. (A) Consistent with Fig 2E., Monkey K showed that the best decoding neuron set has high correlations in tuning diagram between passive and BMI navigation; the correlation drops when including less informative neurons (Mann-Kendall Test, $p<0.01$). However, such trend was not observed in Monkey M. (B) The corresponding decoding performance of (a) relative to the full model as a function of number of neurons.

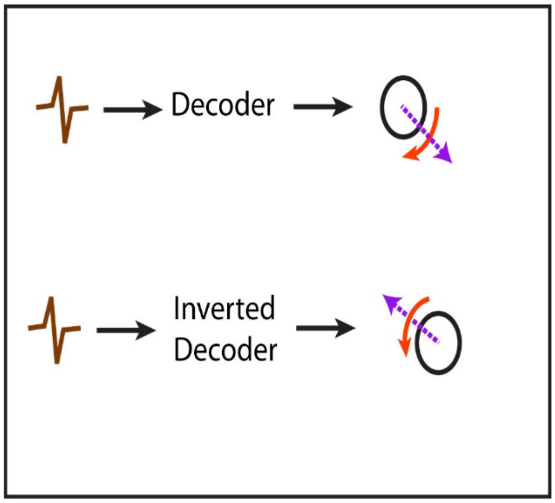

Figure S5. Schematic of decoder output. The decoder was trained from neural activity tuned to velocity in wheel chair coordinates. As an example a regular decoder output would move the wheelchair forward and right but when inverted, given the same neuronal activities it would now move backward and left.

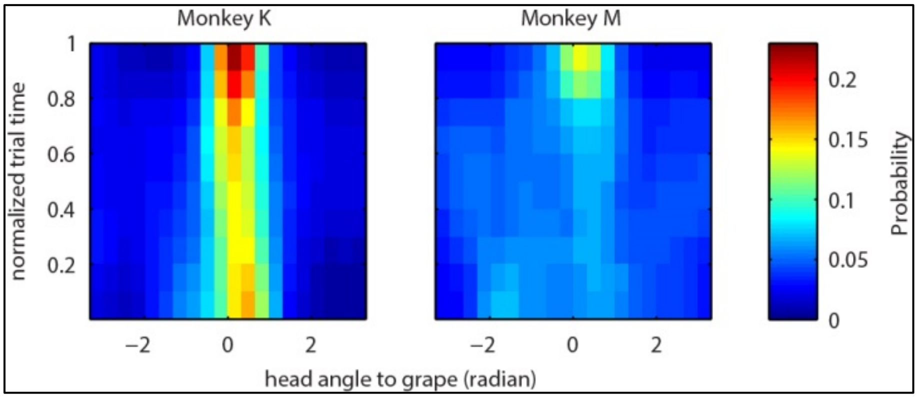

Figure S6. The distribution of head orientation as the function of trial time. The x-axis is the head angle in room coordinates, where zero corresponds to the head pointing to the grape. The y-axis is the normalized trial time, which normalizes the trial time from zero (the beginning of a trial) to one (the end of a trial). The left panel is the distribution for Monkey K, and the right figure is for Monkey M.

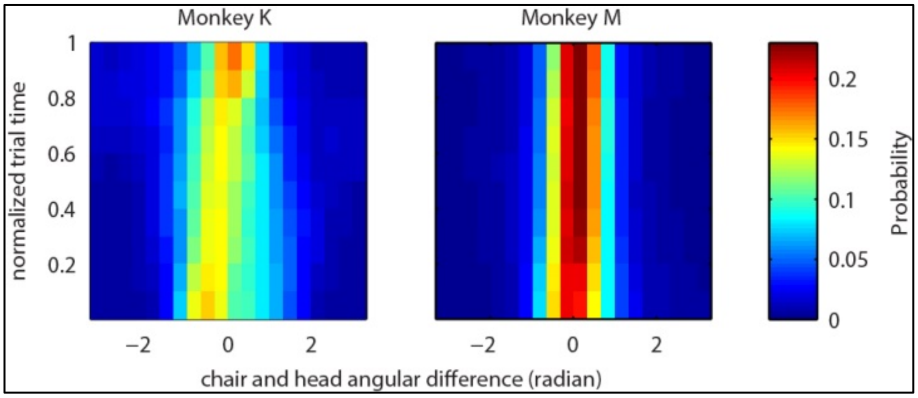

Figure S7. The distribution of the relative orientation between head and wheelchair as the function of trial time. The x-axis is the angle in room coordinates, where zero corresponds to the wheelchair pointing to the same direction as the head. The y-axis is the normalized trial time, which normalizes the trial time from zero (the beginning of a trial) to one (the end of a trial). The left panel is the distribution for Monkey K, and the right figure is for Monkey M.

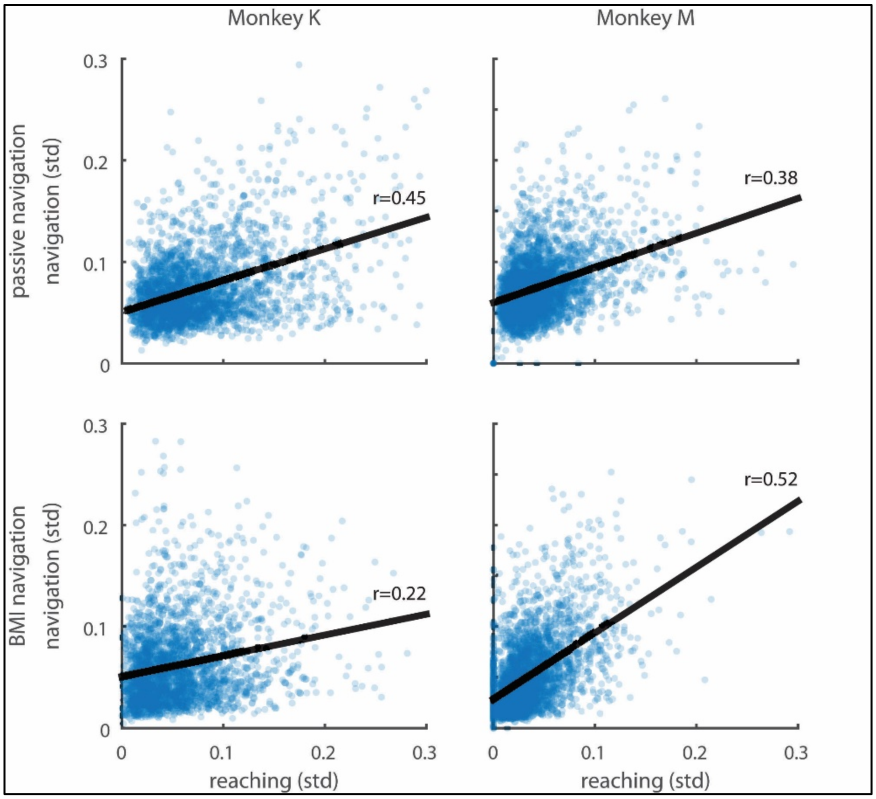

Figure S8. Standard deviations of the normalized firing rates during navigation and reaching. Each dot represents the standard deviation of the normalized firing rate of a neuron in a session. The solid line is the regression line, and the Pearson correlation is shown in the right end of the line (all $p<0.05$).

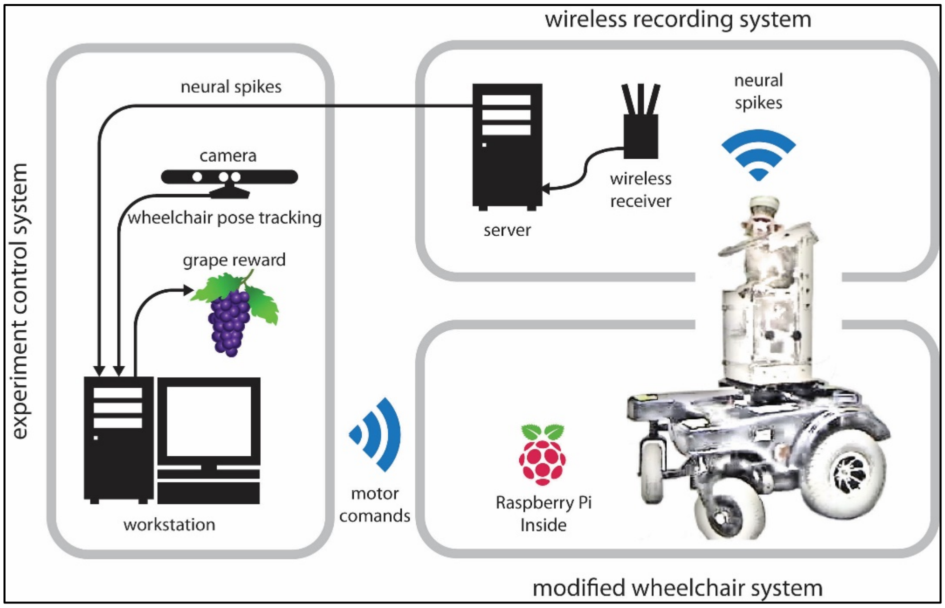

Figure S9. Overview of the BMI navigation system. The driving experiment was composed of three systems. The experiment control system controls the experiment flow, decodes monkey's neural signals, tracks the pose of the wheelchair and delivers the grape reward. The wireless recording system receives the monkey's spiking activities from the monkey's head stage, and sends the activities to the experiment control system. The modified wheelchair system executes the (decoded) wheelchair movement commands from the experiment control system.

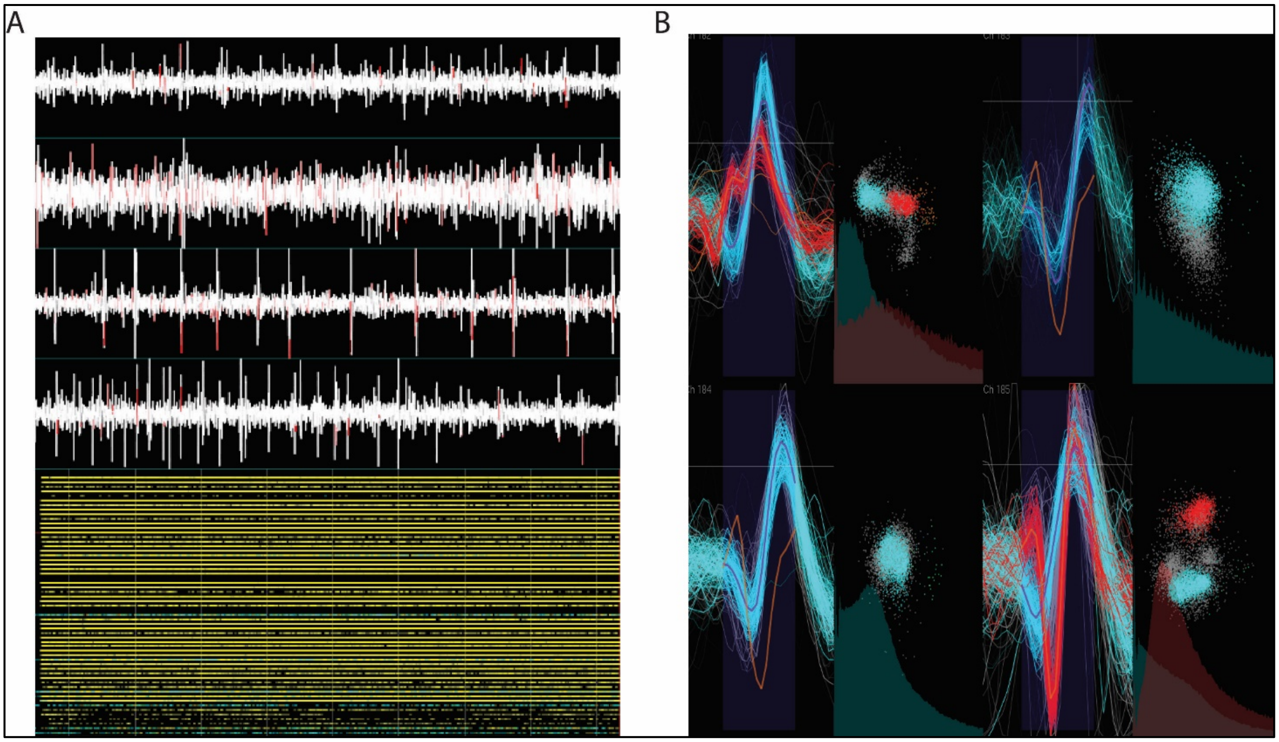

Figure S10. Screenshot of spike voltage traces from four different channels as displayed on the wireless client interface. (A) Raster plot in bottom panel depicts neuronal spiking activity, each row pertains to a single neuron, and each dot is an individual spike, recorded during a session (128 channels displayed) from monkey M. (B) Screenshot of the wireless client with four channels alongside their corresponding PCA clusters and spike templates. Each channel can record a maximum of two neurons.

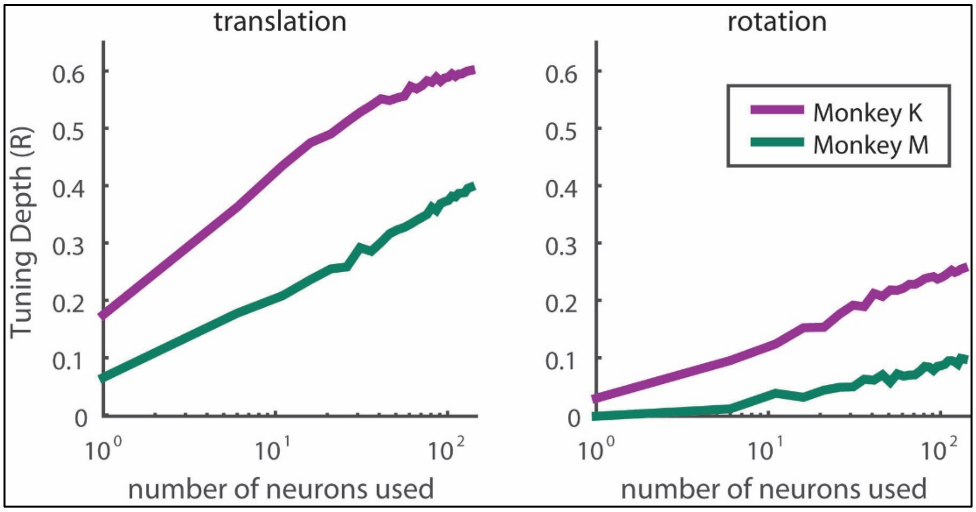

Figure S11. Neuron-dropping curves. Neuron dropping curves are shown separately for decoded translational and rotational velocity commands.

Supplementary Movie Legend

Movie S1. Experiment at a glance

Supplementary Methods

Wireless Recording System

Briefly, the wireless recording system comprised the following: digitizing headstages, a wireless transceiver, a wireless-to-wired bridge, and client software. Four headstages were attached to the transceiver module for a total of 128 channels per transceiver unit on which spike sorting could be performed. The bridge received incoming radio packets and converted the signal to an Ethernet interface that collected in the client computer which could be visualized with the custom designed software client (Fig S10). We recorded from 128 channels in Monkey K and 256 channels in Monkey M, yielding 140 and 144 neurons from Monkey K (79 neurons from M1, 35 from S1, and 26 from Premotor) and Monkey M (72 neurons from M1, and 72 from S1). The number of neurons we acquired from a session was 147.0±10.2 for Monkey K and 157.1±23.9 for Monkey M on average.

Robotic Wheelchair

Our robotic wheelchair was a modified, commercially available mid-wheel drive model (Sunfire General) manufactured by Drive Medical. It was powered by two 12volt, 36Ah batteries connected in series to obtain 24VDC. The motors were 450 watt units and were coupled to each drive wheel through a 1:21 ratio transmission, controlled individually to result in directional and speed control. The electromagnetic brakes were removed since braking was obtained with a two channel motor controller added to the unit. The stock VR2 70A motor controller and joystick controls were also completely removed. The powered wheel chair weighed approximately 200Kg and provided a stable low center of gravity platform with a cruising range of 38.8Km rolling on 12 inch diameter drive wheels.

A Roboteq VDC2450 dual channel motor controller served as the interface between an onboard Raspberry Pi (RP) and the wheelchair motors. The RP received the computed motor commands through User Datagram Protocol (UDP) from the experiment control system, and the RP sent the commands to the Roboteq controller via serial data bus connection. As a safety feature, the Roboteq was programmed to stop the robotic wheelchair when the communication failed (i.e., the robotic wheelchair did not receive any motor commands for 1 s), or hit obstacles (i.e. the wheels failed to turn as current limit was set to 50 Amps). An emergency manual power disconnect was prominently placed on the vehicle that would disable the power to the wheels should a malfunction occur that requires a complete manual shut down. A secondary 2.4GHz wireless control system also interfaced to the Roboteq controller and was used as a remote manual wireless control to assist in maneuvering the vehicle between experiments when it was not receiving commands from the experiment control system.

Grape Dispenser

Black Corinth Champagne wine grapes were selected to be used as a solid fruit reward because of their very small size and consistency. Long duration experiments involving hundreds of reward transactions can be conducted without the animal losing appetite or interest. The grapes were delivered using an in house designed grape dispenser.

A pneumatic dispenser system was designed and built to allow computer control of this reward. The main mechanical parts of the dispenser were a rotating platter disc and stationary mount plate with a single drop chute. The parts were designed using SolidWorks 3D Cad and were CNC milled from solid blocks of aluminum and have an anodized coating for corrosion resistance. Each dispenser consists of 50 chambers of 13mm diameter, 13mm high oriented on a 340cm diameter rotating platter into which the grapes were loaded.

A pneumatic double acting cylinder (Robart model 166) was used to ratchet advance the platter one chamber at a time around an axis exposing the drop chute that directs the grape to a pedestal, presenting the reward. A secondary pneumatic cylinder (Robart model 165) was mounted vertically over the drop chute and was time delay activated to push out any grapes that may stick in the mechanism over the course of the experiments.

The air cylinders were actuated with 100 psi air through directional control valves (Ingersol Rand model P261SS-012-D-M) and equipped with pneumatic speed controls and regulated to permit the pneumatic actions to be tuned for silence and smooth operation. Optoisolators (PVG612) were used to interface the 24volt control pneumatic valve to the experiment control system via a National Instruments data acquisitions unit (NIDAQ). The Optoisolators were mounted onto a custom designed printed circuit board which we use universally for interfacing to various powered actuators in the lab.

Tuning Depth

To obtain the *Tuning Depth* that characterized the representation of velocity by each neuron, the following multiple linear regression was performed

$$n(t) = \sum_{\tau=-10}^{10} \alpha(\tau)V_t(t-\tau) + \beta(\tau)V_r(t-\tau) + \gamma + \epsilon(t)$$

where $n(t)$ is the spike count at time t; τ is the lag of time bins (each bin was 100 ms); $V_t(t)$ and $V_r(t)$ are the translational and rotational velocity command at time t, $\alpha(\tau)$ and $\beta(\tau)$ are regression coefficients;γ is the intercept; and $\epsilon(t)$ is the residual error. Once the regression model was fitted, we calculated the goodness-of-fit (R^2), and the *Tuning Depth* was the square root of R^2. Because the τ ranged from 1 s before to 1 s after the spike count measurement, it allowed the Tuning Depth to capture any tuning before or after that time.

We also investigated the tuning in finer temporal resolution (i.e., at each time lag). We applied the equation above; however, instead of summing over all the time lag τ, each τ was considered separately to obtain *Tuning Depth* for each τ. Therefore, we could learn when the neuron was best tuned to the velocity commands.

This equation could also be used to calculate whether a neuron was tuned to other behavioral variables by simply replacing $V_t(t)$ and $V_r(t)$ by other kinematics measure, such as acceleration.

Neuron dropping curves of the tuning depths were computed for both monkeys across all sessions (Fig S11).

Behavioral Improvement

The performance on each trial was characterized by trial duration and the length of the trajectory from the starting location to the reward location. For each session, the medians of trial duration and trajectory length were obtained to represent the behavioral performance of the session. Next, Mann-Kendall Trend Test was performed on these medians for all the sessions to see whether a trend was present.

Comparison between Decoders across Days

To compare decoding across different experimental days, we applied a decoder trained on day A to neuronal data recorded on day B, and vice versa, and measured the change in decoding performance in passive navigation trials. More specifically, to compare two decoders A and B trained on passive navigation of sessions A and B, respectively, we started with identifying neurons that were found in both sessions. Next, we normalized the firing rate of each neuron by subtracting its mean and dividing by its standard deviation. Then, decoder A was trained on session A, and decoder B was trained on session B. Decoder A was then applied to session A and B, and decoder B was applied to both sessions. This way, for each session, we had two

predictions of velocity during passive navigation: the one obtained using the same day's decoder and the one obtained by a different day's decoder. The comparison of these two predictions, quantified as Pearson correlation, was used as a measure of the decoders' similarity.

Inverted Decoder

After the last session, we ran an additional session to see whether the monkeys learned to utilize the directional properties of the decoder. The hypothesis was that if the monkeys utilized BMI directional commands, their performance would have been significantly impaired after the decoder output was inverted. In this inversion procedure, the absolute values of the translational and rotational velocity commands were kept the same, whereas their signs were reversed: forward movement was turned into backward movement, backward into forward, leftward into rightward, and rightward into leftward. In this control experiment, the monkeys went through 30 trials of passive navigation as usual. Then, the monkey controlled the robotic wheelchair the regular way for the first half of BMI navigations (~45 trials). However, during the second half of BMI navigation (~45 trials), the decoders were inverted.

Offline Wiener Filter Distance Decoding

For each mode (brain-control, passive navigation), Wiener filters were trained and tested using each mode's neural ensemble and kinematics data to decode distance, through five rounds of 2-fold cross-validation, for each session. The R-of-prediction for all five rounds were averaged to obtain the R value for each session. To test the generalization of the filters, for each session we applied all Wiener filters (five per session) trained in one regime to the other's data, and averaged the resulting R-of-predictions to obtain the session's R-value.
